# Electrospun Fiber as a Facile Means of Studying Silver Nanowires under Mechanical Stretching

**DOI:** 10.1002/smsc.202200069

**Published:** 2022-12-01

**Authors:** Jie Huang, Guangyu He, Yuxiong Hu, Yiwei Sun, Dongfu Wang, Zhu-Jun Wang, Xueyang Liu, Hongyu Chen

**Affiliations:** ^1^ Institute of Advanced Synthesis (IAS) and School of Chemistry and Molecular Engineering Jiangsu National Synergetic Innovation Centre for Advanced Materials Nanjing Tech University Nanjing 211816 P. R. China; ^2^ Research Institute of Zhejiang University-Taizhou Taizhou 318000 P. R. China; ^3^ School of Physical Science and Technology Shanghai Tech University Shanghai 201210 P. R. China; ^4^ Department of Chemistry School of Science Westlake University 18 Shilongshan Road Hangzhou 310024 Zhejiang Province P. R. China

**Keywords:** Lomer–Cottrell (LC) locks, silver nanowires, strain hardening, stretching

## Abstract

It is a great challenge to study the mechanical property of nanomaterials because it is difficult to get a hold of them and apply force. A convenient alternative method via electrospun fibers is shown, where silica‐coated fivefold twinned silver nanowires are well aligned in the fiber and the fibers are aligned in the membrane to a similar direction of macroscopic stretching. The product silver nanowires show necks whereas the fractured silica shell acts as the internal marker for the extent of stretching. Surprisingly, the stretched neck length depends on the nanowire diameter but is independent of the degree of stretching, indicating that the necks have arrived at a limiting intermediate state, which is otherwise hard to achieve for nanomaterials. The necks are studied by transmission electron aberration‐corrected microscopy and steered molecular dynamics simulation, revealing that the build‐up of dislocations and stacking faults (the Lomer–Cottrell (LC) locks) are responsible for trapping the necks at similar limiting states.

## Graphical abstract

Silica‐coated fivefold twinned silver nanowires are well aligned in nanofibers by electrospinning, and the subsequent uniform stretching reveals a rare intermediate state, apparently limited by the strain‐hardening effect. Transmission electron aberration‐corrected microscopy and steered molecular dynamics simulation prove that dislocations and stacking faults (the Lomer–Cottrell (LC) locks) are responsible for trapping the necks at such states.

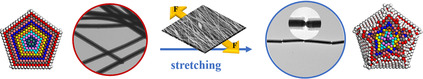

## 1 Introduction

Most of the studies on nanomaterials focus on their photonic,^[^ ^1^ ^]^ electric,^[^ ^2^ ^]^ and magnetic properties,^[^ ^3^ ^]^ with few on the mechanical properties.^[^ ^4^ ^]^ The latter is of equal importance to the former, as both would provide fundamental support for the applications of nanomaterials.

From the historical viewpoint, understanding and manipulating the mechanical properties of materials may lead to greatly improved applications, and even historical milestones, for example, the breakthroughs of bronze and ironworking. Essentially, modern metalworking was made possible with the accumulation of synthetic approaches to improve the mechanical strength and pliability, and reveal the underlying principles.^[^ ^5^ ^]^ It is only when mankind fully understands lattice structure and defects, before we could rationally modulate the mechanical properties, for example, removing defects by annealing, and improving the mechanical strength of steel by minute Fe_3_C defects.

When stretching or bending bulk metal wires, the strain would cause the materials to harden (the strain‐hardening effect). Upon further stretching, the hardened area would form a neck, followed by a fracture. It is generally believed that the hardening is caused by the accumulation of crystal defects, so their interlocking would constrain the overall structural deformation, presenting higher mechanical strength than the segments without stretching. The fracture usually occurs at the neck, which is often the weakest site because the hardening cannot make up for the much‐reduced cross‐section.

At the nanoscale, it is conceivable that the mechanical properties would critically depend on the specific defects such as the fivefold twinning of Ag nanowires (AgNWs), unlike polycrystalline bulk metals with complex boundaries and random defects. However, it is not trivial to stretch nanowires like bulk materials. They are simply too many and too small to manipulate. It has become a major bottleneck in studying the nanoscale mechanical property and in advancing the application of nanomaterials.

A pioneering approach is to use extremely precise equipment, to pick up and hold on to the two ends of a nanowire, so that it could be stretched with in situ monitoring by scanning and transmission electron microscopy (SEM and TEM, the in situ method). Such a process is challenging and costly, typically only applied to a few nanowires in a study. Sliding of the lattice boundary was observed before the breaking of AgNWs, and high‐density dislocations were observed at the fracture surface.^[^ ^6^ ^]^

In contrast, computational simulation (molecular dynamics) could offer structural information at the atomic details, though the size of the system is usually small and limited by the available computational power.^[^ ^7^ ^]^ Typical systems involve thousands of atoms, dealing with nanowires of only a few nanometers in diameter. As such it is difficult to simulate the fivefold twinning observed in typically thicker nanowires (*d* = 40–120 nm). Under stretching, it was observed that dislocations are blocked at the twin boundaries, leading to strain‐hardening effect.^[^ ^8^ ^]^

Another approach is to prepare polymer–nanowire composite films by spin‐coating or doctor‐blading (the membrane method).^[^ ^9^ ^]^ It is usually difficult to apply uniform forces to the nanowires, considering that the nanowires are embedded in the polymer matrix with random orientations, and that the nonuniform membrane would stretch differently under force. Most importantly, such membranes are usually thick (over 20 μm), which causes various complications and makes it difficult to observe the embedded nanowires.

In this work, we show that electrospun fibers are an alternative method for stretching silica‐coated AgNWs, which could be easily incorporated in the polymer fiber with uniform distribution and well alignment along the axis of the fibers. The directional spun fibers could uniformly stretch, and the nanowires could be readily studied by TEM and SEM. Most importantly, our method allows a limiting intermediate state to be preserved and characterized.

## 2 Results and Discussion

**Figure 1:**
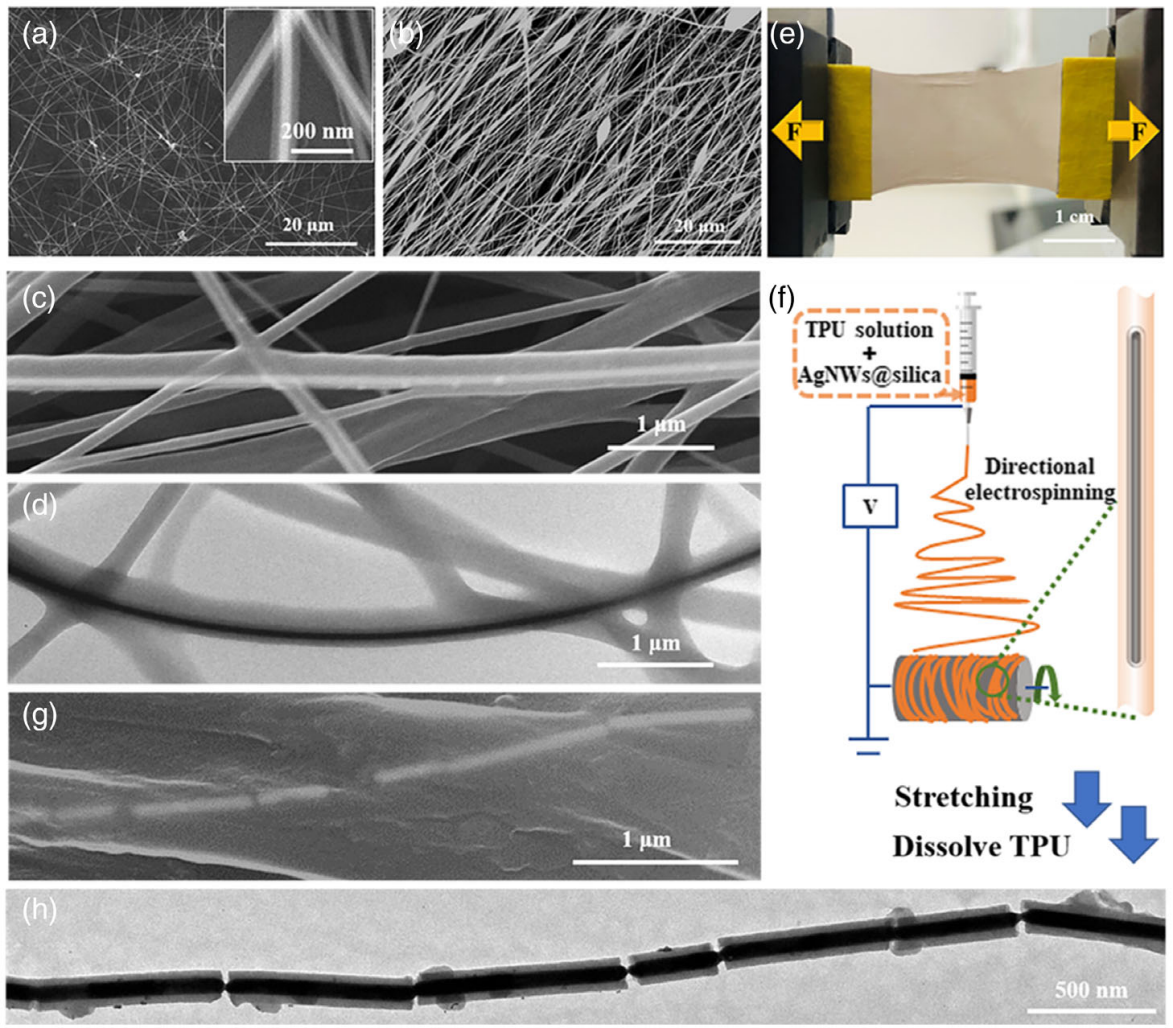
a) SEM image of the AgNW@silica and inset shows the magnified view. b) Typical SEM image of the aligned AgNWs@silica@thermoplastic polyurethane (TPU) nanofibers. c) Photograph of a nanofiber film loaded on the tensile machine. d) Typical SEM image of the aligned AgNW@silica@TPU nanofibers before stretching. e) Schematics illustrating the preparation of AgNW@silica@TPU nanofibers. f) TEM image of the AgNWs@silica@TPU nanofiber. g) SEM image of the AgNW@silica@TPU nanofibers after stretching. h) TEM image of the AgNW@silica after stretching and cleaning.

The AgNWs with fivefold twinning, average length of 40 μm, diameter of 70 nm, and with poly(vinylpyrrolidone) (PVP) as the surface ligand were synthesized via the one‐pot polyol process.^[^ ^10^ ^]^ As reported, the AgNWs are straight with uniform diameter, without kinks or necks. In high‐resolution transmission electron microscopy (HRTEM) images, the twin plane is the most prominent and common feature (Figure S1, Supporting Information). The as‐synthesized AgNWs were isolated from ethylene glycol by centrifugation and re‐dispersed in isopropanol. After the AgNWs were coated with a layer of silica,^[^ ^11^ ^]^ a core–shell nanostructure (AgNW@silica) was obtained and the silica shell appears complete and smooth (**Figure** **1**a), with an average shell thickness of 44 nm.

Subsequently, the AgNW@silica was dispersed with thermoplastic polyurethane (TPU) in *N,N*‐dimethylformamide (DMF) solution, and the mixture was used for electrospinning. Through the high‐speed rotation of the fiber‐collecting roller, the directionally aligned AgNW@silica@TPU fibers formed a membrane. The color of both the AgNW@silica solution and the acquired AgNW@silica@TPU fiber membrane are silverish gray (Figure S2, Supporting Information), reflecting the presence of AgNWs.

From the SEM images, the AgNW@silica@TPU nanofibers are well aligned (Figure 1b), and their average diameter is 230 nm. With a close‐up view, the embedded AgNWs could be recognized with a bright contrast, whereas the silica is indistinguishable from the polymer shell (Figure 1c). Most of the fibers are free of embedded nanomaterials, which is not surprising given the huge weight ratio of the two (about 68:1). In the TEM images (Figure 1d), an obvious three‐layer structure can be seen, where the AgNW shows the darkest contrast. In all of the images we have inspected, the AgNWs remain straight and free of kinks or necks. The silica shell is used to protect and maintain the mechanical stability of the AgNWs during the electrospinning process. Without it, some of the AgNWs were found bent which caused ambiguity in the following stretching.

As a facile tool for embedding nanomaterials in fibers, electrospinning has been extensively studied. To date, nearly all of the attention was on the modified properties of electrospun fibers,^[^ ^12^ ^]^ as opposed to using the fibers for manipulating the embedded nanomaterials. For our purpose, the diameter of the polymer fiber can be controlled at a similar scale of nanowires, and thus the spatial confinement forced the nanowires to align parallel to the direction of the fiber.^[^ ^13^ ^]^ Moreover, the fibers can be arranged in roughly a similar direction through the directional spinning roller receiver. Thus, a two‐level alignment is easily achieved from the nanoscale AgNWs to the bulk scale fiber membrane. This provides a reliable means for indirectly stretching the embedded nanowires.

The AgNW@silica@TPU fiber membrane was trimmed to a 2 × 3.5 cm^2^ rectangle, with the long side aligned to the direction of fiber orientation (Figure S2, Supporting Information). To dislodge the AgNW@silica@TPU fibers from the aluminum foil substrate without stress, they were soaked in an aqueous NaOH solution to dissolve the aluminum. Both ends of the collected fiber membrane are strengthened with glue to a hard aluminum foil (Figure 1e), so that the membrane could be stretched evenly. As shown in Figure 1b, there are some fibers that are not exactly parallel with the stretching direction. With the angle parallel to the stretching direction defined as 0°, 98% of the fibers had an average distribution angle range of ±9°. The small misalignment is not expected to cause significant transverse force during stretching (cos 9° = 0.98).

Upon stretching, the macroscopic tensile force is transmitted to the constituent fibers and then to the internal AgNW@silica, as the fibers are largely independent of each other (Figure 1f). With an equal stretching ratio for the polymer fibers, it is expected that the AgNW@silica would experience similar tensile stress.

It is well known that pure TPU fiber membrane has excellent elasticity, and 200% stretching is well within the range of elastic deformation.^[^ ^14^ ^]^ In addition, TPU is easy to prepare, has a wide range of applications, and is cheap to obtain. Importantly, the carbonyl groups of TPU are expected to interact strongly with the polar Si–OH groups on the silica surface via H‐bonding. It is true that the silica–TPU interaction is much weaker than the metallic bonding within the AgNW, the combined interaction from all surfaces could be stronger than the latter, as clearly shown by our experiments.

In the SEM images, when the AgNW@silica@TPU fibers are stretched more than 145% (we define 100% stretching as the initial nanowire without stretching), cracks appeared on the surface of the loaded fiber membrane (Figure S3a, Supporting Information), indicating significant inelastic deformation. To avoid the uneven stretching caused by cracks, we selected 130% as the maximum stretching for the following study. The minimum stretching degree was 110% of the fiber membrane length, as 105% stretching did not give the multi‐neck structures. To prevent complications caused by fiber bouncing back, the stretched membrane was maintained the same length by adhering to a conductive tape.

When observed by SEM, there were no cracks on the loaded fiber membrane under 130% stretching (Figure S3b, Supporting Information), but the AgNWs embedded in the fibers were obviously broken (Figure 1g). With a thick TPU shell, it was difficult to observe the internal AgNW@silica. Thus, the fibers were dissolved in DMF to remove the TPU coating, and the remaining AgNW@silica was isolated for TEM characterization.

In the TEM images (Figure 1h), the AgNWs were still covered with a silica shell. Obviously, the silica shell is less ductile than the internal metal nanowire, with the latter stretched longer into “necks” whereas the former showed a clean‐cut fracture surface. As such, the gap in the silica shell (the silica gap) serves as a convenient internal marker for the extent of the stretching in each case. The locations of the necks appeared quite random, but the gap sizes have a narrow distribution around 30–40 nm (vide infra).

In our survey of 73 AgNWs after 130% stretching, the average length (2.4 μm) is significantly shorter than the initial 40 μm. 45 (62%) of them had no neck in the middle; 22 (30%) showed a single neck; 6 (8.2%) showed more than one neck. Among the first category, most of the free ends were the fractured end without a silica cap, with only a small percentage (1.3%) showing the original core–shell structure. The latter two categories included 9 (12%) of broken‐but‐aligned cases: The two fractured ends are separated by a short distance, but the nanowire segments are still aligned (Figure S4, Supporting Information). It is probably broken during the drying step; otherwise, there is no way that two separated colloidal pieces could still meet each other with perfect alignment. Thus, it appears that the aforementioned ratios of the single‐ and multi‐neck nanowires are significantly underestimated due to the breaking of AgNWs.

**Figure 2:**
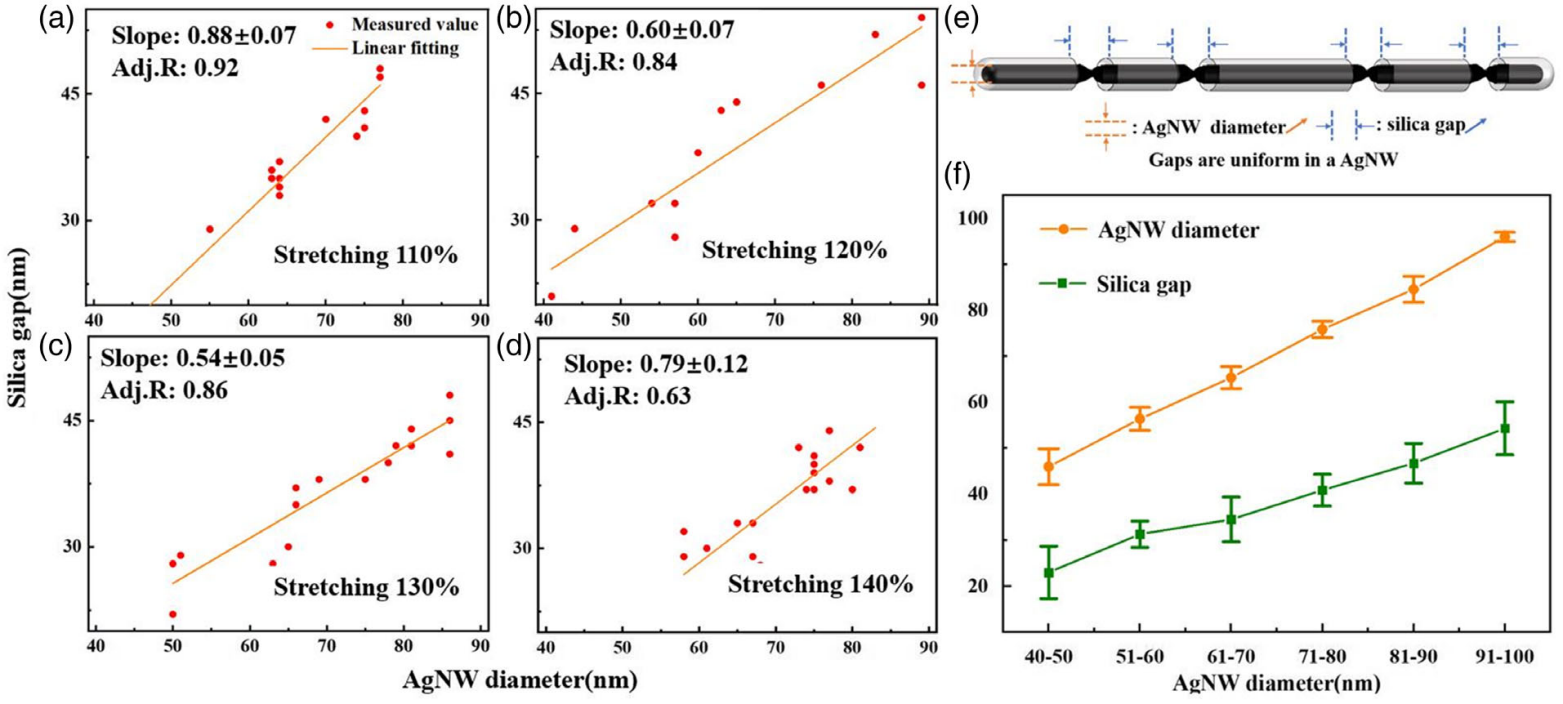
a–d) Plots showing the dependence of silica gap size on the specific AgNW diameter, with stretching degrees of 110%, 120%, 130%, and 140%. e) Schematics illustrating the definitions. f) Plot showing the narrow distribution of silica gap size and its dependence on the AgNW diameter.

**Figure** **2**a–d shows the dependence of silica gap size on the AgNW diameter. In the samples with different stretching degrees (110%, 120%, 130%, and 140%), the silica gap size always increased with the AgNW diameter. The narrow distribution of the results validates our method for studying the mechanical properties of nanowires. The underlying trend conforms to the general rule of “smaller is stronger”^[^ ^15^ ^]^ for typical nanomaterials. In other words, thicker AgNWs could be stretched further, showing higher ductility.^[^ ^6^ ^]^ In the literature, it is known that ductility is a different concept from tensile strength, and that it usually takes a stronger force to stretch a thinner nanowire.^[^ ^16^ ^]^

All these data of different stretching degrees are grouped together, and recategorized according to the AgNW diameter (Figure 2f). It shows that the silica gap has a very small length distribution, regardless of the stretching degree. Hence, the observed necks are likely in a limiting intermediate state: As the AgNW is stretched to form necks, the strain‐hardening prevents further stretching and thus all necks arrive at the same gap size. Our new method makes it possible to survey a large number of necks and understand their dependence, unlike conventional studies that are often case‐specific.

Such a limiting intermediate state is a unique advantage. In the in situ method, it is a great challenge to precisely modulate the force applied, such that all necks would arrive at similar hardened states. More often than not, the stretched states last for only a fraction of a second,^[^ ^17^ ^]^ making it extremely difficult to fully characterize the intermediate lattice structure. To the best of our knowledge, the only previous method that could produce a static intermediate state is by stretching the glass‐coated Pt electrode.^[^ ^18^ ^]^ The resulting structure is likely equilibrated at the high temperature (430 °C) and removing the thick and strained glass shell would be nontrivial.

For macroscopic materials, the strain‐hardening effect is well known. For example, when bending a steel wire repeatedly, the first bend hardens and thus the second bending site would usually occur slightly off at a nearby location, and so on. When stretching a metal wire, the strain‐hardening effect is caused by the accumulation of defects, and there are typically five stages: elastic deformation, plastic deformation, hardening, necking, and fracture.^[^ ^19^ ^]^ It should be noted that there is usually only one neck or breaking site in each wire, for both macroscopic^[^ ^16^ ^]^ and microscopic^[^ ^6^ ^]^ systems. Multiple fractures for a wire only occurred when the wires are embedded in a polymer/silica matrix.^[^ ^9^, ^18^ ^]^ It is possible that the tensile stress is well distributed in the matrix during the indirect stretching, so that the strain is shared among the multiple segments.

**Figure 3:**
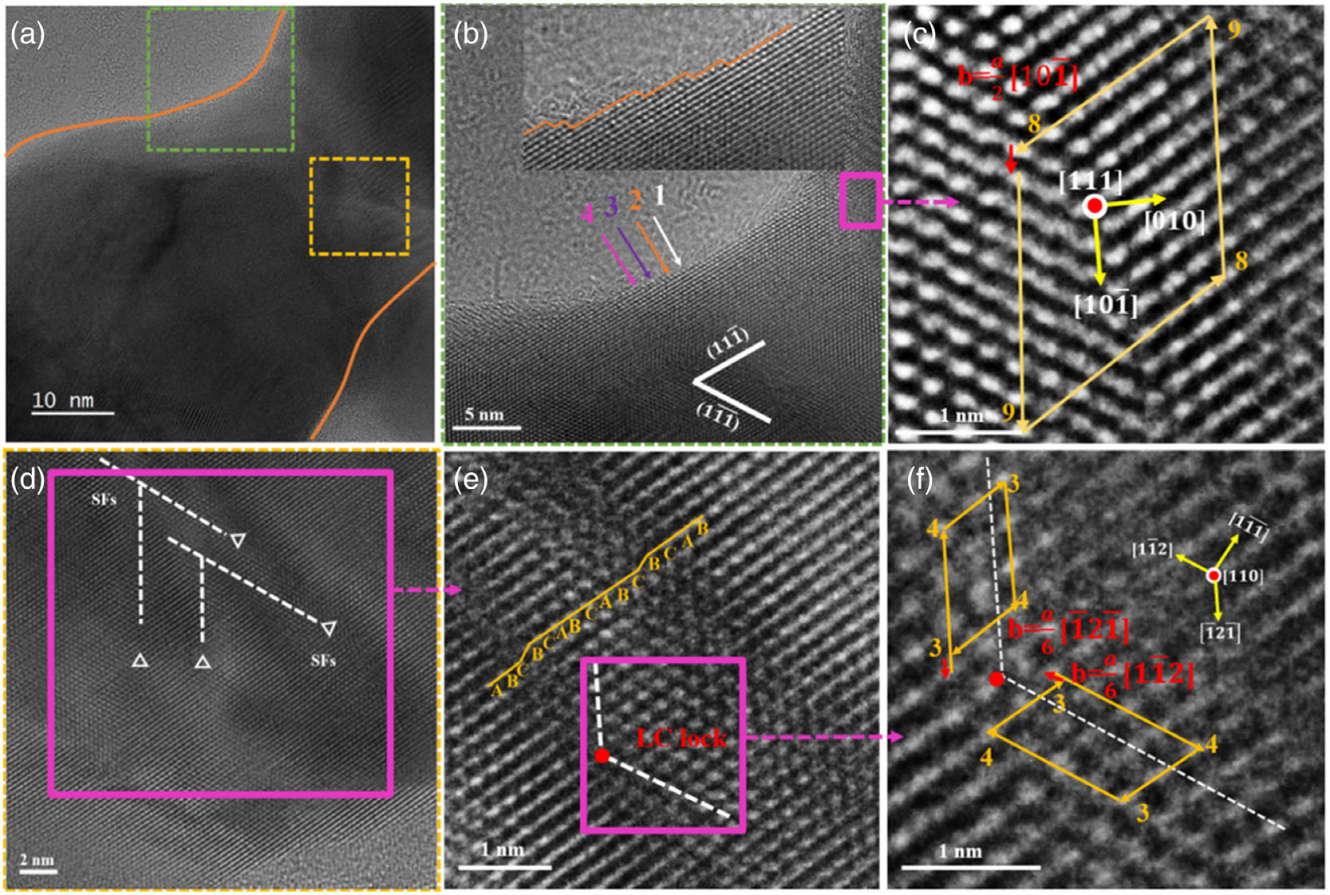
a) HRTEM of the neck region of a typical stretched AgNW. b) AC‐TEM image showing the surface steps along the {111} surface planes in the neck region (the green box in (a)). c) AC‐TEM image showing the enlarged view (the pink box in (b)) of the dislocation. d) AC‐TEM image showing the enlarged view (the yellow box in (a)) of hierarchical stack fault networks (white dashed line) and e) enlarged view of the pink box in (d) showing the Lomer–Cottrell (LC) locks (red dot) in different intersecting {111} slip systems and a representative stack fault marked by the ABCBCABCABCBCAB‐stacking sequence. f) Further enlarged view of the pink box in (e) showing the Shockley partial dislocation.

To characterize the neck structure in detail by high‐resolution TEM (HETEM), all surface layers have to be removed: The AgNW@silica@TPU fibers were dissolved in DMF to remove TPU and then in 90 °C NaOH solution to remove the silica shell. At low magnification, it can be seen that most part of the AgNWs remained intact with the initial flat facets, except that the neck region has become thinner with a smooth concave surface. **Figure** **3**a shows the neck region of a single‐neck AgNW. Despite the significant shape change, the distorted neck still showed two sets of lattices in selected‐area electron diffraction (SAED) (Figure S5, Supporting Information), the same as the initial AgNWs with fivefold twinning. It shows that the twin planes have not been destroyed, though the scattering intensity did become weaker suggesting disruptions.

Figure 3b shows the aberration‐corrected transmission electron microscopy (AC‐TEM) images with atomic resolution, where the surface steps are clearly visible. The smooth concave curvature at the neck is favorable (energetically less costly) in terms of sliding atomic metal planes.^[^ ^20^ ^]^ It is incompatible with flat facets and thus the formation of surface steps provides the necessary means to create a smooth curvature. In other words, the neck surface reflects the low‐energy kinetic pathway upon mechanical stretching, not the flat facets with thermodynamic stability.

Dislocations and stacking faults are known to occur when the lattice is not perfectly aligned, which is expected when the {111} planes of AgNWs are tilted and pulling against each other. As expected, many kinds of lattice dislocations could be found in the neck region. Clear unit dislocations were found (Figure 3c), and high‐density of stacking faults on the {111} sliding planes gave a network structure (Figure 3d). By labeling atomic sequences, a typical stacking fault is shown in the enlarged view (Figure 3e).

**Figure 4:**
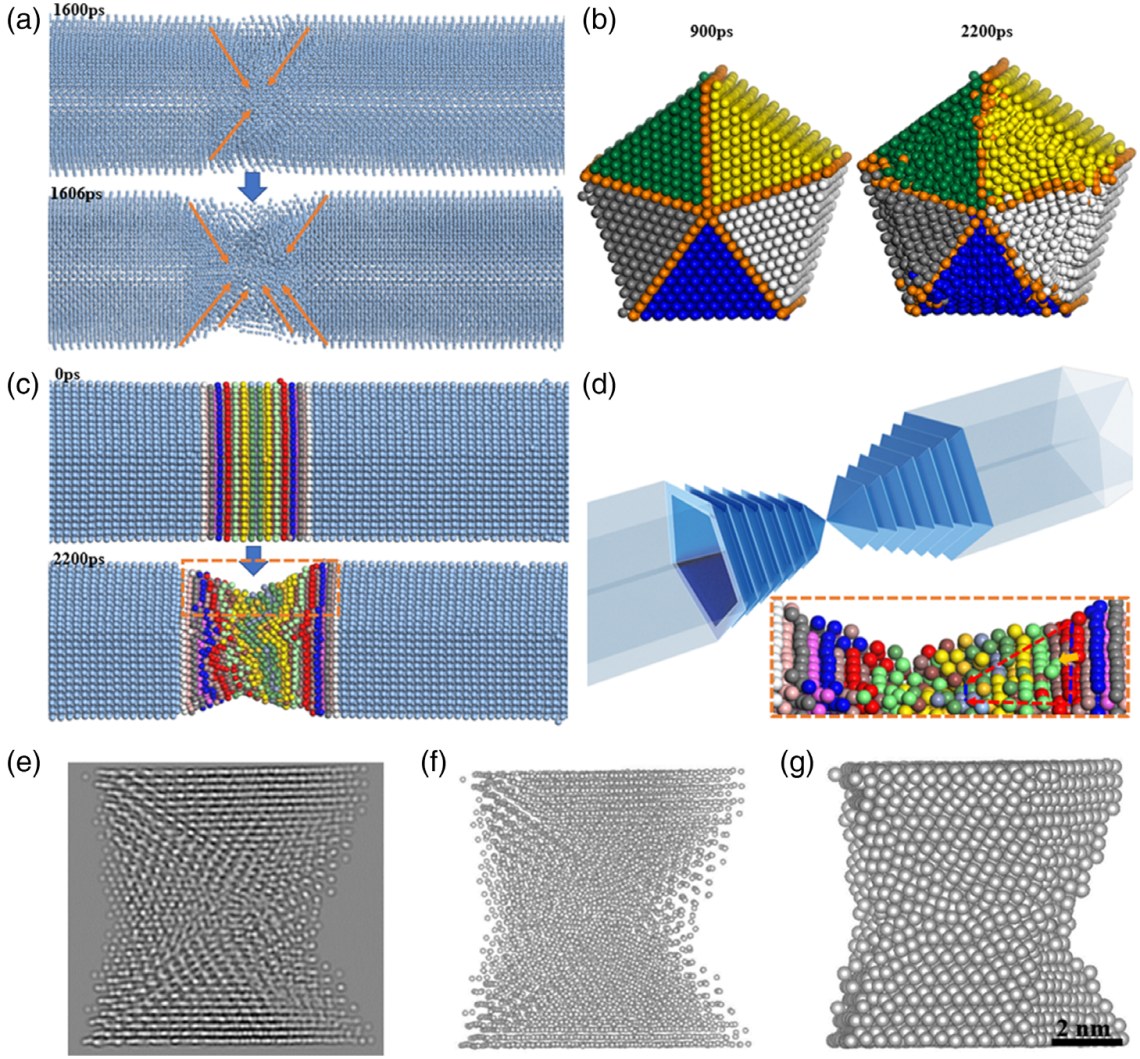
Different coloring schemes are used to illustrate the atomic movements. a) Semitransparent view showing the inward sliding of the {111} planes that allow the subsiding of the middle segment, and the lengthening of the core region, at the moment of neck formation (1600–1606 ps). b) Each single‐crystalline domain of the fivefold twinning AgNW model is marked with different colors, and the orange atoms represent the grain boundaries. The cross‐section of the neck region before (at 900 ps) and after stretching (2200 ps). c) Each layer of atoms is marked with different colors to illustrate the movements in the neck region, showing the initial nanowire (0 ps) and the nanowires after stretching (2200 ps). d) 3D schematics showing the sliding {111} plane, and the inset shows the enlarged view of the orange box in (c), showing the top edges of the {111} planes. e–g) The images of Q‐STEM simulation (e), ball‐and‐stick model (f), and space‐filling model (g) of the neck region. See the Supporting Information for an all‐around rotating video of these models.

For metals with face‐centered‐cubic (FCC) lattice, {111} planes are the most closely pack planes with the highest binding energy and thus, the most common slide planes. In the process of AgNW stretching, the neck becomes longer with the inward sliding {111} planes (**Figure** **4**a). Parallel sliding of the {111} planes is not feasible because of the fivefold twinning. As the cross‐section becomes smaller, each layer of the {111} plane is sequentially smaller (Figure 4d).

In the AC‐TEM images, we first determined the ($11\bar{1}$) plane according to the crystal plane spacing, and then the other crystal orientations are obtained by their relative angles. Among the stacking faults slide along the {111} planes, Shockley partial dislocations with $\frac{a}{6}\left[1\bar{1}2\right]$and $\frac{a}{6}\left[\bar{1}2\bar{1}\right]$ Burgers vectors were marked. For lattice materials, dislocations are known to move during the plastic deformation, such that a perfect $\frac{a}{2}\left[110\right]$ unit dislocation tends to dissociate into a pair of $\frac{a}{6}\left[112\right]$ Shockley partial dislocations,^[^ ^21^ ^]^ the latter of which was identified (Figure 3f). In comparison to the direct movement of dislocations with a large barrier, these partial dislocations can move more easily in materials.

In our system, the {111} slip system is not isolated, but moves together in a group in the fivefold twinned AgNWs. When the partial dislocations from the different planes meet on the intersection line, a complex dislocation is formed containing three partial dislocations and two stacking faults. It is called Lomer–Cottrell (LC) locks,^[^ ^22^ ^]^ which should explain the strain‐hardening effect in our system.

To better understand the formation of LC locks and to visualize real‐time atomic movements, we constructed a simplified AgNW with fivefold twining through molecular dynamics (MD) simulations. The axis of AgNW is set at [110], which is parallel to the {100} surface. The diameter is 3 nm and the length is 25 nm, involving a total of 34 408 Ag atoms. The large gap defect is not presented in the model, similar to the literature studies.^[^ ^6^, ^7^, ^8^ ^]^ The AgNW was stretched through steered molecular dynamics (SMD), where one end was fixed and the other stretched by a constant 1 m s^−1^ velocity^[^ ^23^ ^]^ along the axis direction. Necking occurs at 1600–2200 ps and the neck continues to stretch thinner, until the nanowire fractures at 4036 ps.

Different coloring schemes were used to highlight the structural changes: 1) As the neck region lengthens, the extra central core is constructed via the sliding of {111} planes (Figure 4a). 2) There was little mixing among the five single‐crystalline domains after the stretching (Figure 4b, Figure S6, Supporting Information), which is remarkable considering the severe structural changes. It explains why the twin planes are still retained in the neck and the domains are independent of each other in terms of generating defects. 3) As the {111} planes slide toward the central core, more edges of the internal layers are exposed, forming steps at the outmost surface. As shown in Figure 4c, the combination of the {111} plane top edges is not {111} plane. They become a smooth curve in a larger system as characterized in Figure 3b. 4) Each {111} plane is a triangle, and it becomes sequentially smaller in the shrinking cross‐section of the neck (Figure 4d and inset). The excessive atoms are forced to move along the twin planes and crowded around the central core, such that the boundary atoms move relatively faster, forming a sharp angle pointing at the center of the neck (Figure 4c).

While the simulation model shows all atomic details in 3D space, the HRTEM only shows the 2D projected atomic images along the *Z* direction of the electron beam. On the basis of the MD simulations at 1900 ps, we simulate the projected diffraction images at different angles via quantitative scanning transmission electron microscopy (Q‐STEM, Figure 4e and Figure S8a, Supporting Information) and Visualization for Electronic and STructural Analysis (VESTA, Figure 4f,g and Figure S8b,c, Supporting Information). The video in Supporting Information shows the all‐around views of the 3D models, confirming a high degree of matching among them. Rather than a few surface atoms, the simulated diffraction pattern represents the majority of the atoms, and thus, the orderly fringes suggest that the neck region still has a semi‐crystalline structure (not amorphous) despite the numerous defects therein. The slanted (111) fringes are consistent with our hypothesis.

**Figure 5:**
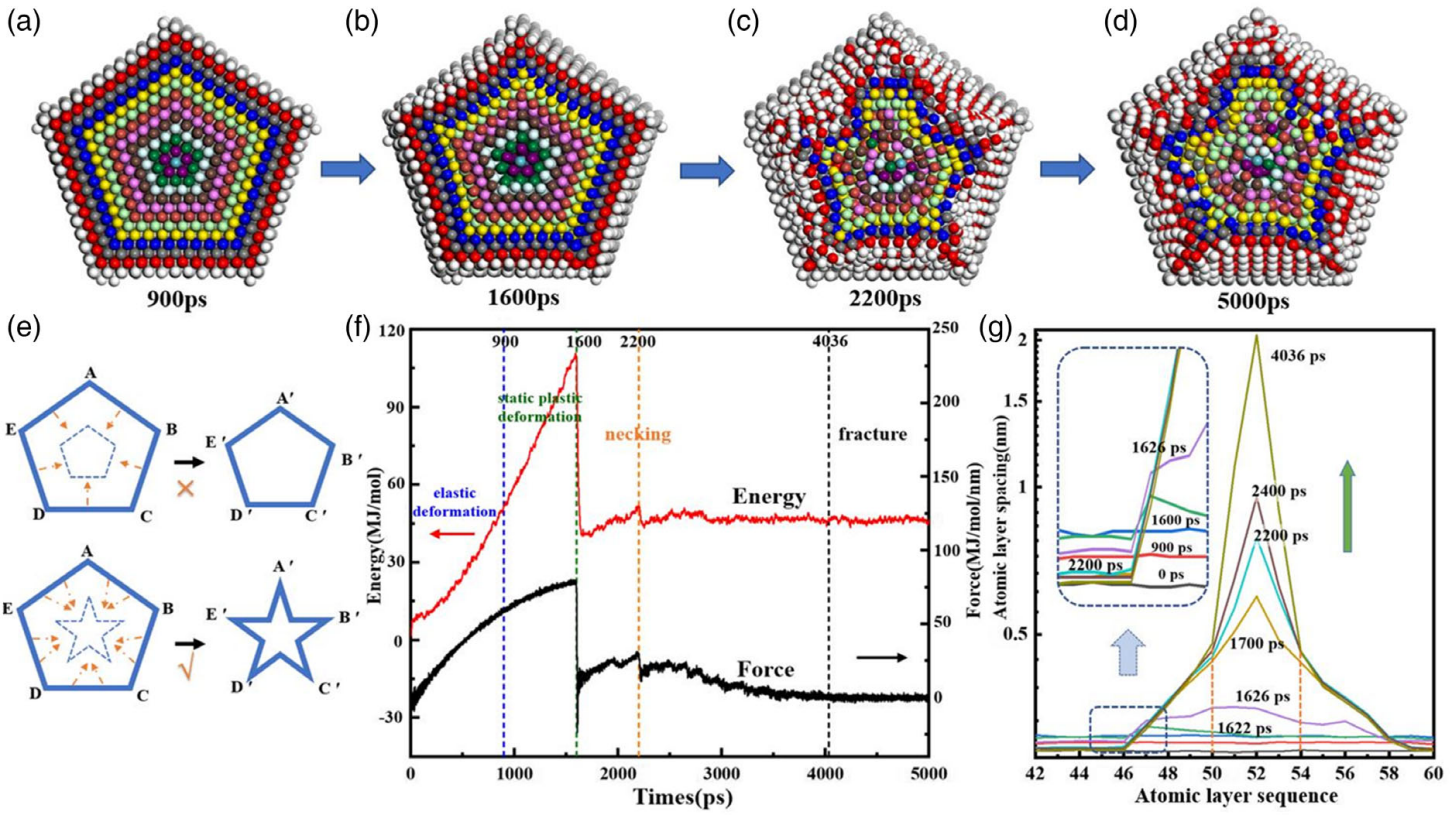
a–d) Sectional view of the neck at four time points: a) 900 ps, b) 1600 ps, c) 2200 ps, d) 5000 ps. e) Schematic diagram of unit dislocation decomposes into partial dislocation. f) Energy barrier and force diagram of AgNW during stretching. g) The average distance among the atoms (no longer the bond lengths after the initial movements) of the neighboring {110} planes changed with time.

From the cross‐sectional view (**Figure** **5**a), as the {111} planes move inward and become smaller, the excessive atoms build up near the grain boundaries. The necking starts at 1600 ps (Figure 5b), and the product at 2200 ps (Figure 5c) shows a characteristic star shape, with the retained twin boundaries going through the star tips. The inner layer of atoms near the central core is more disrupted than the outer layers. In Figure 5d, it can be seen that even after the AgNW is completely broken at 5000 ps, there is no further advancement of the concave borders of the star shape from the cross‐sectional view, consistent with the behavior of the LC lock. (Panoramas of the AgNW at 900, 1600, 2200, and 5000 ps in Figure S7, Supporting Information)

The process from the pentagon to star shape in the neck cross‐section is a manifestation of dislocation decomposition on atomic movement (Figure 5e). The origin of two partial dislocations marked in Figure 3f can be deduced from Thompson tetrahedron: $\frac{a}{2}\left[101\right]=\frac{a}{6}\left[211\right]+\frac{a}{6}\left[1\bar{1}2\right]$and $\frac{a}{2}\left[\bar{1}10\right]=\frac{a}{6}\left[\bar{1}2\bar{1}\right]+\frac{a}{6}\left[\bar{2}11\right]$. From the energy point of view, the combination: $\frac{a}{6}\left[1\bar{1}2\right]+\frac{a}{6}\left[\bar{1}2\bar{1}\right]\to \frac{a}{6}\left[011\right]$ satisfies the energy condition $\sum {\left|b_{before}\right|}^{2}>\sum {\left|b_{after}\right|}^{2}$.^[^ ^16^ ^]^ The new dislocation $\frac{a}{6}\left[011\right]$ has^[^ ^11^ ^]^ Burgers vector and the slip surface is {001}, which is not the closely packed plane in FCC crystal. Therefore, this dislocation cannot slip, thus forming an LC lock.

Figure 5f shows the temporal change of energy and force. There is a major upward rise before the neck formation that starts at 1600 ps. With the plane slipping and bond stretching occurring at the neck region, the energy and force then undergo a sharp drop. Measurement of the average distance among the atoms of the neighboring {110} planes (Figure 5g) shows a continuous increase of bond distances until the necking starts, and the bond lengths at the intact regions then return to the initial length, agreeing well with the sharp drop of the energy/force curves.

The 2nd kinetic barrier occurs at 2200 ps, likely because of the strain‐hardening effects of LC locks. The physical lockdown is supported by the similarity of the atomic pattern at 2200 ps and after fracture (Figure 5c,d). For the neck region undergoing structural deformation, the average atomic distance no longer reflects the bond lengths, but the extent of deformation. After 2200 ps, the changes mainly occur for the atoms between the 50th and 54th layers. In other words, the central neck region is made by the downward sliding of a few layers in the middle as shown in Figure 4a.

In our system, the formation of multiple necks in an AgNW could be due to the distributed strain in the stretched matrix (TPU), but the fact that the necks (silica gaps) have very similar lengths suggests that they are all hardened to a similar degree, likely due to similar LC locks. Fortunately, our method of stretching could trap the limiting intermediate state for detailed analysis.

## 3 Conclusion

We have used electrospun fiber as a facile means of stretching the embedded AgNW@silica. It allows the survey of a large number of stretched AgNWs to reveal their common characteristics. Most importantly, our method creates a limiting intermediate state that is nearly impossible to create by single‐nanowire manipulations via the in situ method. Detailed studies by AC‐TEM and MD simulation reveal that the LC locks are responsible for the strain‐hardening effect in our system, locking all necks of the AgNWs at similar states.

The defects in polycrystalline bulk materials are complex and random, whereas those in nanostructures are highly ordered, such as the fivefold twinning along the axis of AgNWs. Manipulating and understanding the behavior of these orderly defects under mechanical stress would offer fundamental knowledge of mechanical properties for future applications. Our method would offer an alternative approach to the in situ method, to open a window for fundamental insights.

## Conflict of Interest

The authors declare no conflict of interest.

## Supporting information

Supplementary Material

## Data Availability

The data that support the findings of this study are available from the corresponding author upon reasonable request.
