# Supplementary material for: Electrospun Fiber as a Facile Means of Studying Silver Nanowires under Mechanical Stretching
Source: Small Sci. 2022 Dec 1;3(1):2200069. doi: 10.1002/smsc.202200069 (PMC11935933; doi:10.1002/smsc.202200069)
Supplement: Supplementary file 1 — Supplementary Material [file SMSC-3-2200069-s001.pdf]

Supporting Information

Electrospun Fiber as a Facile Means of Studying Silver Nanowires under Mechanical Stretching

Jie Huang, Guangyu He, Yuxiong Hu, Yiwei Sun, Dongfu Wang, Zhu-Jun Wang, Xueyang Liu,* Hongyu Chen**

Experimental Section

Materials

All chemical reagents were used as purchased without further purification. Silver Nitrate ($AgNO_3$, 99.9%,) and iron chloride hexahydrate ($FeCl_3 \cdot 6H_2O$, 98%) were purchased from Sigma-Aldrich. Poly(vinylpyrrolidone) ($(C_6H_9NO)n$, (PVP, Mw~1,300,000) was purchased from Alfa Aesar. Thermoplastic polyurethane (TPU, 85A) was purchased from SKChemicals Co., Ltd, with 85A hardness. Tetraethyl orthosilicate (TEOS) were purchased from Aladdin. N,N-dimethylformamide (DMF), Ethylene glycol ($C_2H_6O_2$, AR), NH3·H2O (AR grade, 25-28% w/w) and sodium hydroxide (NaOH) were all purchased from Sinopharm Chemical Reagent Co. Ltd. Acetone (C_3H_8O, 99.5%) was purchased from Shanghai Lingfeng Chemical Reagent Co., Ltd. Copper specimen grids (300 mesh) with formva/carbon support film were purchased from Beijing Zhongjingkeyi Technology Co. Ltd. Deionized water (resistance > 18.2 $M\Omega \cdot cm^{-1}$) was used in all reactions.

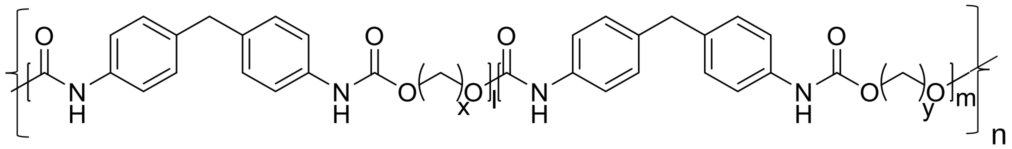

TPU

Characterization

TEM images were collected from a Talos L120C model transmission electron microscope operated at 120kV. Field emission scanning electron microscope (SEM) images were collected on a FEI Quanta 250 FEG model. HRTEM and TEAM images were collected from a JEOL Grand ARM 300F high resolution transmission electron microscope operated at 300kv.

Synthesis of AgNWs：

0.300 g of PVP and 0.200 g of $AgNO_3$ were dissolved in 50 mL of ethylene glycol. A small amount of $FeCl_3.6H_2O$ (0.100 mg, 12.5 μM) was added, and the mixture was stirred at room temperature until it was fully dissolved. Then, the solution was transferred to an oil bath and reacted at 130 °C and 800 rpm for 8 h.

The as-synthesized AgNWs were cleaned twice with acetone:

First, 5 mL of acetone was added to 1 mL of the as-synthesized AgNW solution, and the mixture was shook up and down, let stand, and removed of the supernatant. Then, 3 mL of acetone was added to the AgNWs again, and the mixture was shook up and down, let stand, and removed of the supernatant. Finally, the obtained AgNWs were dispersed in ethanol and used as a stock solution.

Preparation of AgNWs@silica:

400 μL AgNWs(4 mg/ml, H_2O) solution were add to 1 mL isopropanol solvent and stir evenly, and then 40 μL $NH_3{\cdot}H_2O$ and 2 μL TEOS were added. And the solution was stirred at room temperature for 2 h.

Fabrication of AgNWs@silica@fiber:

Take 20 mL of the prepared AgNW@silica concentrate to 350 μL by centrifugation, then mix it into 1.2 mL TPU (26 wt%) solution and stir well. Subsequently, the solution was loaded into a syringe pump and electrospinning was performed by applying 8.5 kV at a pump feed rate of 0.3 mm min^{-1} at an electrode distance of 15 cm, collection time is 160min.

Modeling

All simulations were carried out using a GROMACS software package (version 5.0.7).[1, 2] The fivefold twins AgNWs was described by the gromos53a6 All-Atoms force field[3] and the 12-6 LJ parameters were provided by Hendrik Heinz etc. which reproduced mechanical properties in good qualitative agreement with experiment under ambient conditions for face-centered cubic metals.[4] A rectangular box (12 nm × 12 nm × 50 nm) was applied in our simulations, and the time step was set as 2 fs. The cut-offs of the van der Waals (vdW) interaction was set as 1.2 nm. The temperature of the system was kept at 300 K by the velocity-rescale Thermostat method.[5] The fivefold twins AgNWs were stretched through steered molecular dynamics (SMD). Both ends of fivefold twins AgNWs with 1.2 nm thickness were fixed and stretched by a constant velocity along z direction inversely. To balance the consumption of calculation and accuracy of result, the velocity was set as 1 m/s recommended by the investigation of Zhan and Gu.[6]

The fivefold twins AgNWs were first dealt with NVT relaxation at 300 K for 50 ps to get equilibrated configurations. Then the fivefold twins AgNWs were stretched through SMD at 300 K for 20 ns and Visual Molecular Dynamics (VMD) graphics software was used to inspect the progress of stretch.[7]

Model simulation

The model for simulation is modified by Materials Studio, A cell of 60 Å × 60 Å × 60 Å (x × y × z). From the opening source VESTA (http://jp-minerals.org/vesta/en/download.html), get screenshot images of the ball model and the space-filling model from 0 to 90 degree per 1 degree. Stack these images and output video one and video two by Fiji (https://imagej.net/imagej-wiki-static/Fiji).

The Q-STEM is opening (https://www.physik.hu-berlin.de/en/sem/software/software_qstem) which uses the multi-slice algorithm. The simulation used the TEM mode and Ncells mode. There are 100 slices along Z-axis where each slice is 0.5759 angstrom and potential offset for X, Y and Z are -1, 2 and -1, respectively. In sample tilt area, X is 90 and Z is 0, changing Y from 0 to 90 per 1 for each image. The simulation result is not very precise for the experiment. So, the defocus and Cs were set as zero for the best performance of the model structure. Besides, the

focal spread is set as five nanometers. The images from Q-STEM are .img file, so which were processed by DigitalMicrograph, DM, (https://www.gatan.com/cn/installation-instructions) to get .tiff images. The images from DM possessed equal height and different width whose partial edge area were cut by Photoshop. Stack these images and output video three by Fiji. Combine video one, two and three by Fiji to get the ultimate result.

References:

1. Abraham, M. J.; Murtola, T.; Schulz, R.; Páll, S.; Smith, J. C.; Hess, B.; Lindahl, E., *SoftwareX* **2015,** *1-2*, 19-25. DOI doi.org/10.1016/j.softx.2015.06.001.
2. Berendsen, H. J. C.; van der Spoel, D.; van Drunen, R., *Comput. Phys. Commun.* **1995,** *91* (1-3), 43-56. DOI doi.org/10.1016/0010-4655(95)00042-E.
3. Oostenbrink, C.; Villa, A.; Mark, A. E.; Van Gunsteren, W. F., *JOURNAL OF COMPUTATIONAL CHEMISTRY* **2004,** *25* (13), 1656-1676. DOI 10.1002/jcc.20090.
4. Heinz, H.; Vaia, R. A.; Farmer, B. L.; Naik, R. R., *J Phys Chem C* **2008,** *112* (44), 17281-17290. DOI 10.1021/jp801931d.
5. Bussi, G.; Donadio, D.; Parrinello, M., *JOURNAL OF CHEMICAL PHYSICS* **2007,** *126* (1). DOI 10.1063/1.2408420.
6. Zhan, H. F.; Gu, Y. T., *Comput. Mater. Sci.* **2012,** *55*, 73-80. DOI 10.1016/j.commatsci.2011.12.024.
7. Humphrey, W.; Dalke, A.; Schulten, K., *J. Mol. Graph* **1996,** *14* (1), 33-8, 27-8. DOI 10.1016/0263-7855(96)00018-5.

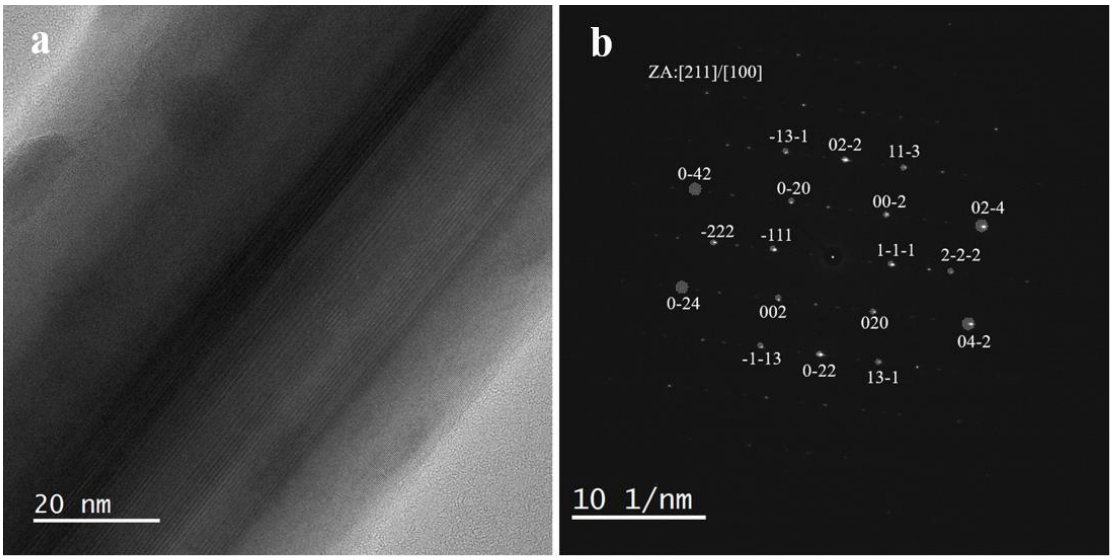

Figure S1. (a) HRTEM image of the fivefold twins AgNW. (b) Indexed SAED pattern of fivefold twins AgNW shows in (a) recorded with low index zone axes of [211] and [100] with respect to two of the fivefold twined domains in the NW. The superposition of the two reciprocal lattices and the {111} fringes in the SAED is characteristic of the fivefold twins AgNW structure. Measured interplanar spacings: d $1\bar{1}\bar{1}$= 0.230, d $2\bar{2}\bar{2}$ = 0.120, d 020 = 0.196, d $04\bar{2}$ =0.091 d $0\bar{2}2$ = 0.140, d $1\bar{3}1$= 0.123.

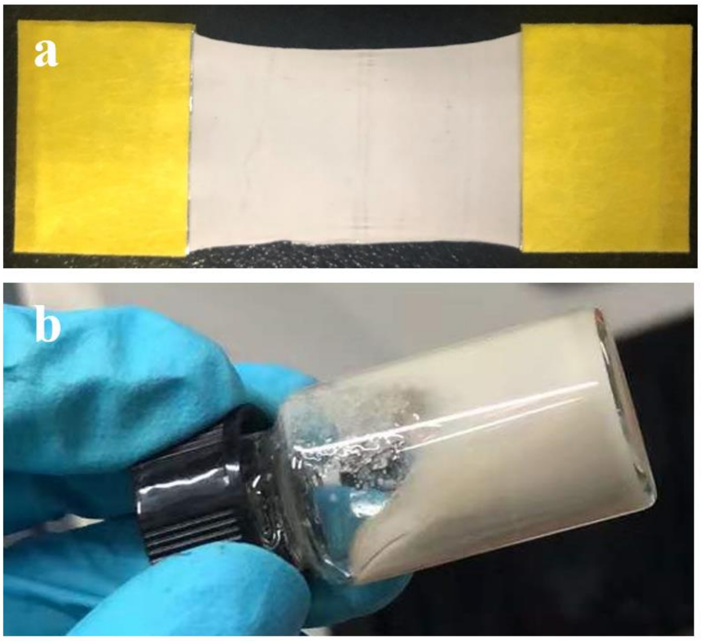

Figure S2. (a) The color of electrospun fiber membrane. (b) The color of Ag@silica@TPU solution.

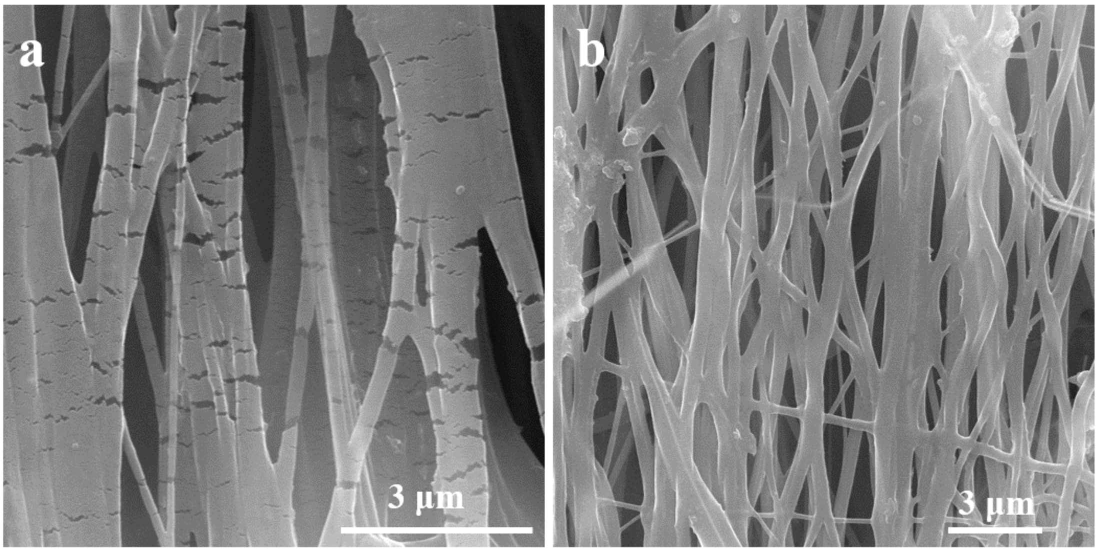

Figure S3. (a) Electrospinning fiber membrane stretching to 145%. (b) Electrospinning fiber membrane stretching to 130%.

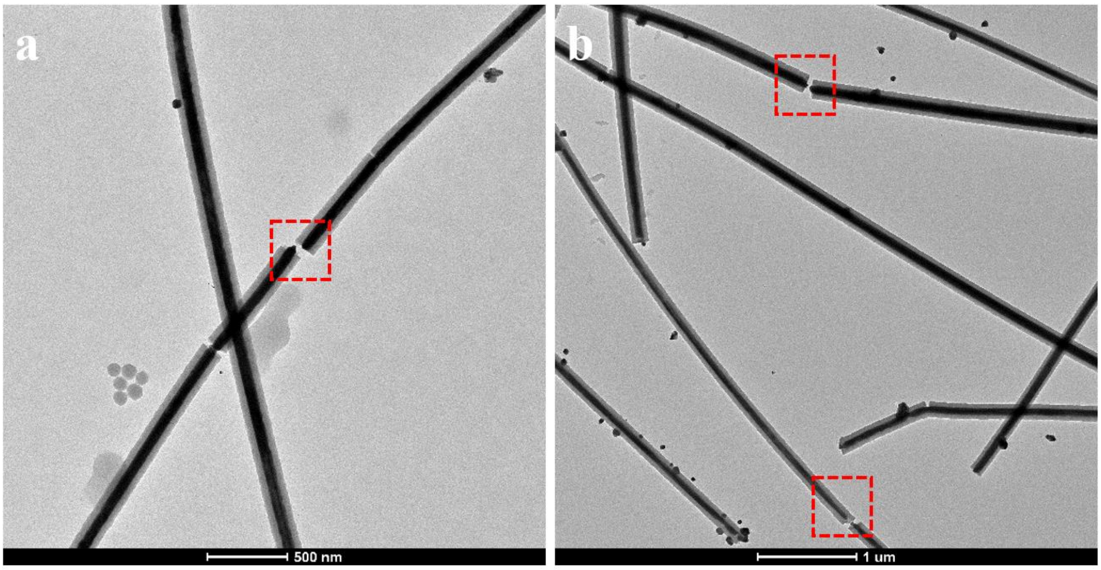

Figure S4. The two fractured ends are separated by a short distance, but the nanowire segments are still aligned.

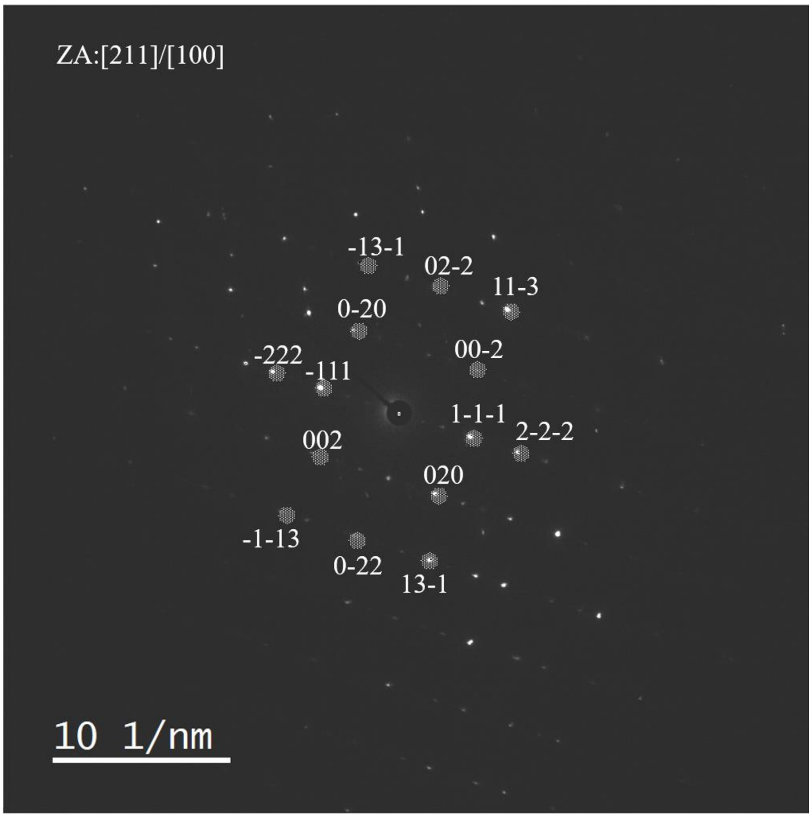

Figure S5. Indexed SAED pattern of neck area.

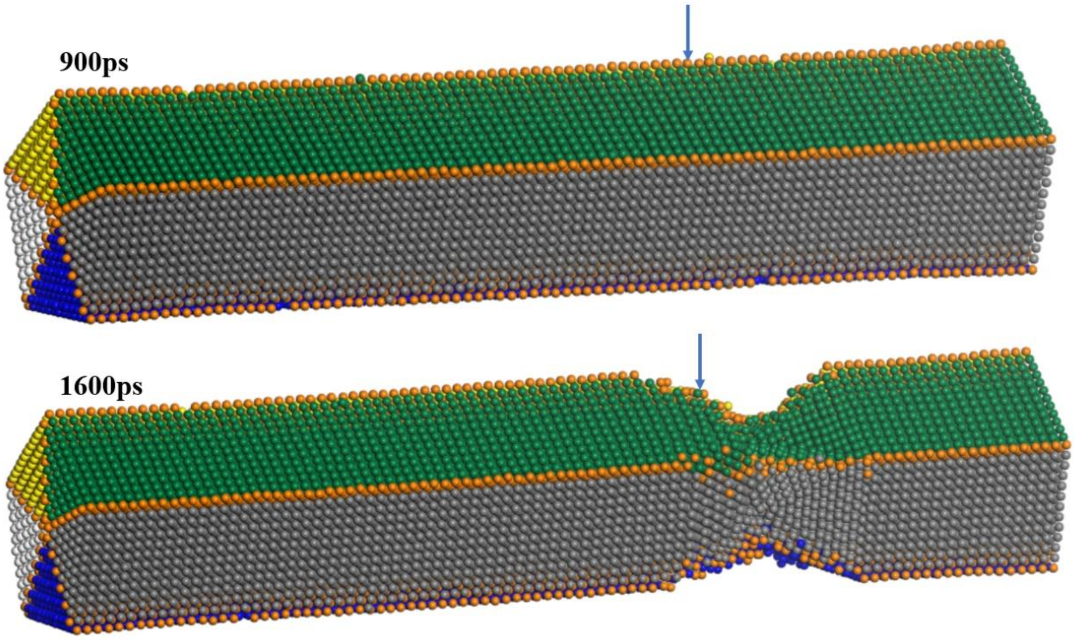

Figure S6. Each single-crystalline domain of the five-fold twinning AgNW model is marked with different colors and stretched, the panorama of the AgNW at 900 ps and 1600 ps, and the blue arrow is the observation point of the model section.

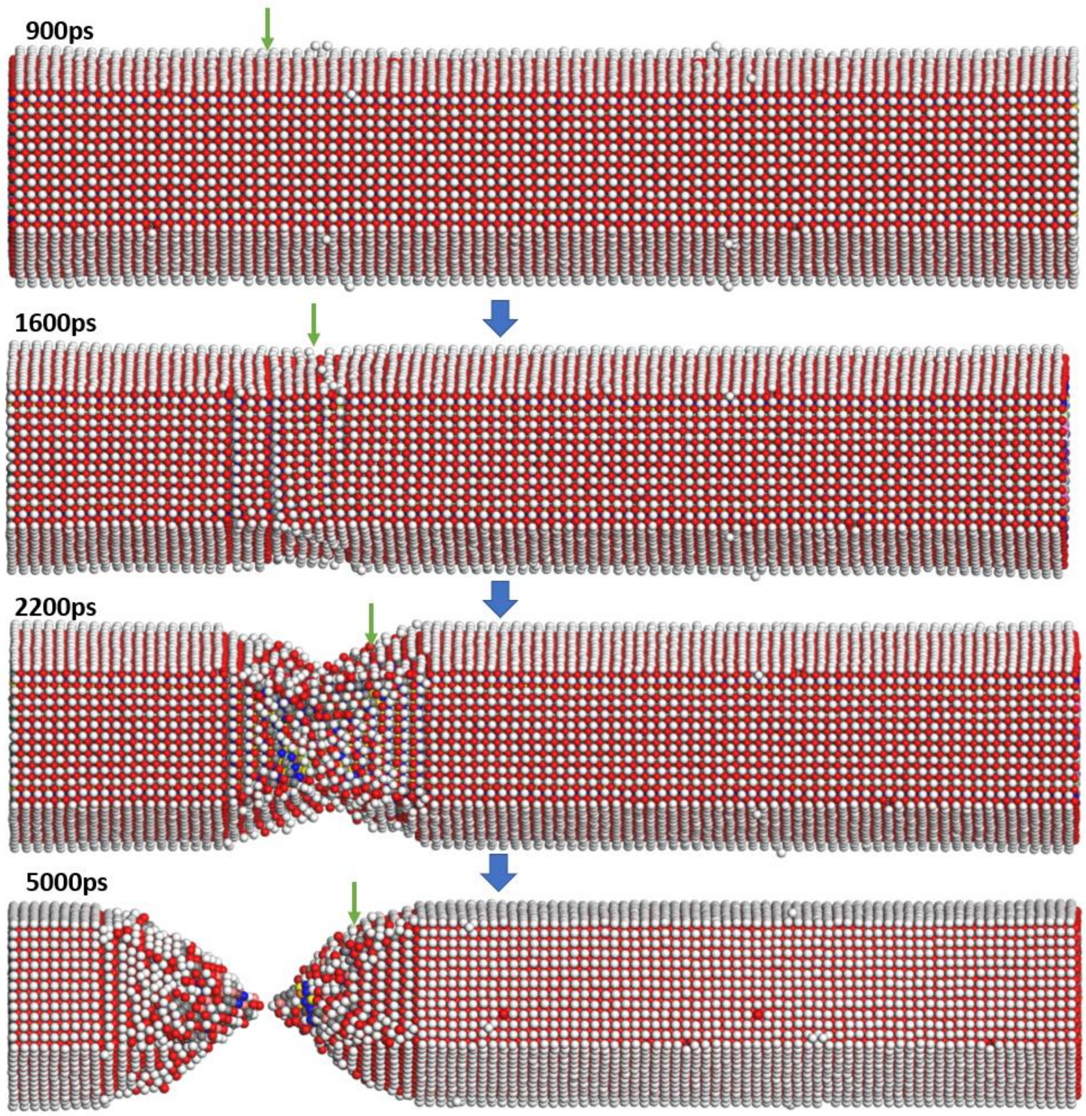

Figure S7. The AgNW with different colors is marked from the central axis atoms to the outermost atoms. The panorama of the model at 900ps, 1600ps, 2200ps, 5000ps. The green arrow is the observation point of the model section.

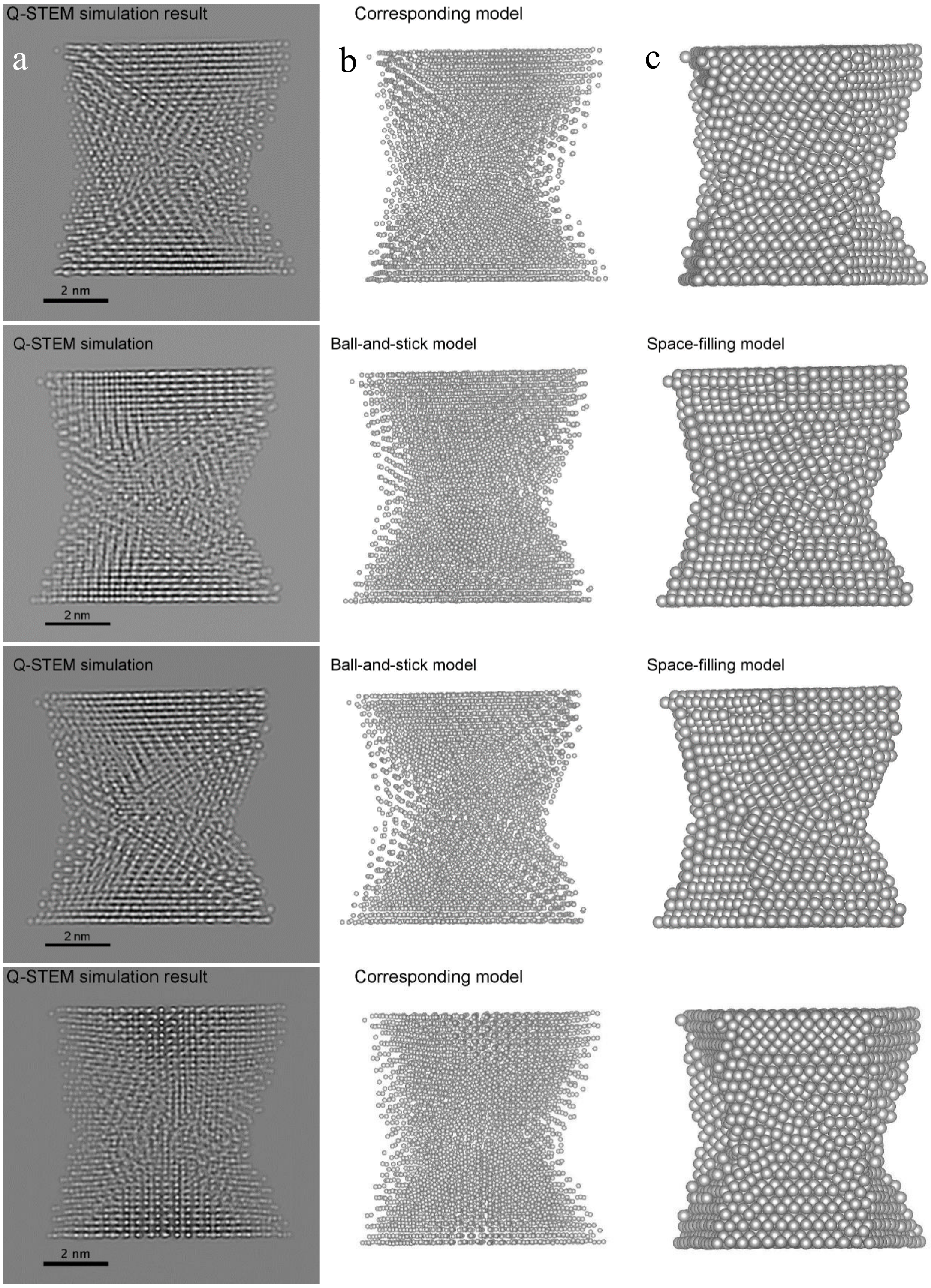

Figure S8. The image of (a) Q-STEM simulation, (b) ball-and-stick model, (c) space-filling model.
